# The willingness for a change

**DOI:** 10.1186/1749-8090-8-150

**Published:** 2013-06-10

**Authors:** Haralabos Parissis, Urszula Simonouk, Bassel Al-Alao

**Affiliations:** 1 Cardiothoracic Dept, Royal Victoria Hospital, Grosvenor Rd, Belfast, BT12 6BA Northern Ireland, UK

**Keywords:** Cardiac surgery, Structural valve degeneration, Bioprosthetic valves

## Abstract

Routine and protocols in our daily practice provide us safety and standardization. However the current knowledge is changing fast and protocols are there to be challenged. This article aims to outline the fine line between skepticism and “constant unbiased interpretation” of the new coming quidelines; the article also suggests a critical eye in data interpretation of the constantly changing technology and its implementation. Finally, by giving examples of the “swing” of the current practice we aim to stimulate awareness.

## Introduction

The ability to question and redefine, ones practice and change it is an obstacle. We come across it daily, with our stubbornness to avoid deviating our routine, to “hold on” to what we clearly know, without necessarily challenging it.

### The stagnation that comes with the lack of scientific approach

It was once said, by the physician Oliver W. Holmes [1], *“I firmly believe that if the whole material medica, as now used, could be sunk to the bottom of the sea, it would be all the better for mankind-and all the worse for the fishes*.”

“Materia medica” is a term used to define the body of collected knowledge about the therapeutic properties of any substance used for healing. We would currently call this Latin terminology “drugs & medical regimes”.

The above quotation was used by the prominent physician [1] and writer of the 19^th^ century. The motive for the above quotation was based at the fact that on the 19^th^ century medicine was looking to define its existence. The most of the breakthrough discoveries were waiting to be made; Homeopathy was blossoming. Pharmacology had an unclear role.

The first half of the nineteenth century and the most intensive first phases of industrialization and urbanization were obviously accompanied by hardship and severe health problems for large parts of the European population. According to Szreter [2] this coincides with a significant decline in life expectancy in Great Britain.

Dr. Holmes was the first to publish recommendations regarding hygiene in hospitals [3]. He prudently identified the significance of hand washing amongst the healthcare workers.

The 20^th^ century witnessed enormous changes in the context of health care. At the beginning of the twentieth century the most important health problems were those associated with the deaths of infants and children and the consequences of infectious disease [4]. In the developed world, these problems have receded, and much more attention is now devoted to the prevention and treatment of chronic conditions and the prolongation of life at older ages.

### Current examples requiring clarification

The medical innovation nowadays is prompting cardiac surgery to follow new avenues; however skepticism in implementation of new ideas and technology is still apparent: for example despite the compelling evidence that complex triple vessel disease is better treated surgically, unessesery ad hoc PCI is still performed in up to 30% of the cases [5]; furthermore despite widespread reports of “two mammaries being better than one” in a cross-sectional observational study analyzed data on 541 368 coronary artery bypass graft, BIMAs are only used in 4% of the cases [6].

Lets take the opportunity and examine the literature that compares all the long-term (15 years plus) studies on the bioprosthetic valves.

David et al. [7] reports freedom from structural valve degeneration (SVD) in accordance with Edmunds definition. The authors brought the patients back every two years for echo follow up.

By enlarge; freedom from SVD is a stricter test than freedom from re-operation, due to SVD. A patient may have SVD but not a re-operation. In David’s paper, 87 patients were reported as having SVD; only 74 patients had a re-operation. Valfre et al. [8] reports freedom from all cause re-operation, which again is stricter than freedom from re-operation due to SVD.

Many papers have follow up times of 5 or 6 years. However, 5–6 years is not a long enough period to detect SVD. Data with such follow up times could be misleading. Yankah et al. [9] reports 20-year data with a follow up time of 4 years and reports the highest rates of SVD out of all valves by a considerable margin. Should this study have had a longer follow up time, the SVD rates would have been far more accurate.

McClure et al. [10]. concludes that pericardial valves are more durable, based on a meta-analysis. However, he does not include the none-composite porcine valves in his analysis.

Lastly, the reason why many surgeons believe pericardium is more durable is because it is so often compared to composite porcine valves, in which case pericardium is more durable. However, in none-composite valves the stress load is reduced because the valve leaflets mimics nature. Reduction of stress reduces calcification.

## Conclusion

“Egocentric type” medicine, whereby the decision making process is taken without considering evidence base, should be avoided. Protocols and Guidelines should be critically scrutinized. The weight should be placed more towards translational research by means of bringing innovation into the daily clinical practice.

And finally, lets accept the healthy criticism and be willing to change: lets manage the uncertainties of practices based on low level of evidence, through making surgical applications more scientific, rational and objective.

“The wind and the waves are always on the side of the ablest navigators”

E. Gibbon

## Abbreviations

PCI: Percutaneous Coronary Intervention; BIMAs: Bilateral Internal Mammary Arteries; SVD: Structural Valve Degeneration.

## Competing interests

The authors declare that they have no competing interests.

## Authors’ contributions

HP conceive the idea and wrote the manuscript; US participated in the design and contributed in reviewing the literature and also helped in the development of the manuscript. BA undertook a literature review and made valuable changes. All authors read and approved the final manuscript.
